# Arsenic resistance strategy in *Pantoea* sp. IMH: Organization, function and evolution of *ars* genes

**DOI:** 10.1038/srep39195

**Published:** 2016-12-14

**Authors:** Liying Wang, Xuliang Zhuang, Guoqiang Zhuang, Chuanyong Jing

**Affiliations:** 1 State Key Laboratory of Environmental Chemistry and Ecotoxicology, Research Center for Eco−Environmental Sciences, Chinese Academy of Sciences, P.O. Box 2871, Beijing 100085, China; 2 University of Chinese Academy of Sciences, Beijing 100049, China

## Abstract

*Pantoea* sp. IMH is the only bacterium found in genus *Pantoea* with a high As resistance capacity, but its molecular mechanism is unknown. Herein, the organization, function, and evolution of *ars* genes in IMH are studied starting with analysis of the whole genome. Two *ars* systems - *ars1 (arsR1B1C1H1*) and *ars2 (arsR2B2C2H2*) - with low sequence homology and two *arsC*-like genes, were found in the IMH genome. Both *ars1* and *ars2* are involved in the As resistance, where *ars1* is the major contributor at 15 °C and *ars2* at 30 °C. The difference in the behavior of these two *ars* systems is attributed to the disparate activities of their *arsR* promoters at different temperatures. Sequence analysis based on concatenated ArsRBC indicates that *ars1* and *ars2* clusters may be acquired from *Franconibacter helveticus* LMG23732 and *Serratia marcescen*s (plasmid R478), respectively, by horizontal gene transfer (HGT). Nevertheless, two *arsC*-like genes, probably arising from the duplication of *arsC*, do not contribute to the As resistance. Our results indicate that *Pantoea* sp. IMH acquired two different As resistance genetic systems by HGT, allowing the colonization of changing ecosystems, and highlighting the flexible adaptation of microorganisms to resist As.

Arsenic (As) is a toxic element present in many environmental biotopes. Inorganic As exists primarily in two valence states: As(III) and As(V). To resist the disruptive effects of As, microbes have evolved a variety of mechanisms, including As(III) oxidation through the activity of As(III) oxidase and As methylation by methyltransferase. Microorganisms can also utilize As in metabolism either as a terminal electron acceptor in dissimilatory As(V) respiration or as an electron donor in chemoautotrophic As(III) oxidation. Nevertheless, the most universal and well-characterized As resistance mechanism is induced by the *ars* system^1^.

The content and organization of the *ars* system vary greatly between strains. Most of the core genes in *ars* operons contain *arsR, arsB* and *arsC*, and other genes are also reported, such as *arsA, arsD, arsT, arsX, arsH*, and *arsN*^2^. A common organization of the *ars* cluster is *arsRBC*^3^, whereas duplicate *ars* operons can also be found in a single strain, such as *Pseudomonas putida* KT2440^4^. Interestingly, in this case more than one *ars* cluster with different structures is observed in the same strain. In all, the content and organization of *ars* operons exhibit great diversity and complexity, and subsequently contribute to the As resistance capability of strains as summarized in Table S1.

The complexity of the *ars* system in diverse bacteria raises the question of its origin and evolution. Different evolution theories have been advanced for the evolutionarily old proteins, efflux pump protein (ArsB) and As(V) reductase (ArsC). For example, ArsB and ArsC may have evolved convergently, as evidenced by sequence analyses^5^. In contrast, *arsC* genes are reported to have a common origin and may have been transferred to other domains by HGT in early times, followed by subsequent divergence to the current phylogeny^6^. Follow-up studies suggest that the HGT events of *ars* genes may be common in nature^7^.

*Pantoea* is a genus of Gram-negative, facultative anaerobic bacteria. This genus belongs to gamma *Proteobacteria*, family *Enterobacteriaceae,* and was recently separated from the genus *Enterobacter*. Currently, the genus contains twenty-six species (http://www.bacterio.net/pantoea.html). Members of this genus are found in various environmental matrices^8^,^9^. In 2013, strain *Pantoea* sp. IMH was isolated for the first time from As-polluted groundwater and reported to resist high concentrations of As, up to 150 mM As(V) and 20 mM As(III)^10^. However, the hyper As-resistance strategy employed by *Pantoea* sp. IMH remains unclear.

Herein, we present the first study of the molecular mechanism of As resistance in strain *Pantoea* sp. IMH. Two different *ars* systems - *ars1 (arsR1B1C1H1*) and *ars2 (arsR2B2C2H2*) - were identified as being responsible for As resistance, contributing in different ways under changing temperature. In addition, we determined that the *ars* genes in IMH were probably acquired by HGT. The insights gained in this study improve our understanding of the flexible adaptation of microorganisms to resist As.

## Results

### As resistance systems in *Pantoea* sp. IMH

**Figure 1:**
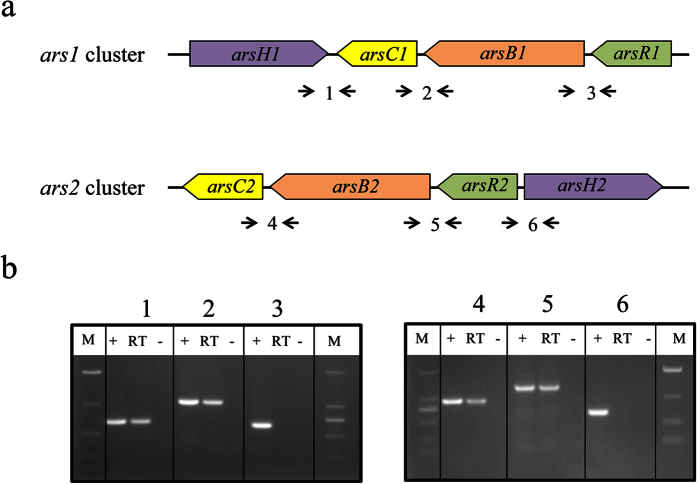
Analysis of co-transcript unit in the *ars* clusters of *Pantoea* sp. IMH by RT-PCR. (**a**) Map position of ars genes and the primers for RT-PCR analysis. Primers used and amplified products (numbered) are given below the schematic representation of the genes. **(b)** Result of RT-PCR reactions with RNA from IMH grown in 1 mM As(V) condition. The numbering on the top of the gels corresponds to the product numbers drawn schematically in the outline given above. M, DNA mark; (+), positive control in which genomic DNA was used as template in the RT-PCR; RT, standard RT-PCR reaction; (−), negative control in which no reverse transcriptase was added to the RT reaction.

Strain *Pantoea* sp. IMH was able to resist up to 150 mM As(V) and 20 mM As(III), whereas *E. coli* W3110 with an *arsRBC* operon did not survive at concentrations above 50 mM As(V) and 5 mM As(III) (Fig. S1). To explore the molecular basis for its hyper-resistance to As, we determined the genome sequence of IMH and identified eight *ars* genes, including two *arsR* encoding a self-repressed transcriptional regulator, two *arsB* encoding a membrane-bound transporter that extrudes As(III) out of the cell, two *arsC* encoding a cytoplasmic As(V) reductase, and two *arsH* encoding an NADPH-dependent FMN reductase with an unknown biological function. These *ars* genes were organized as an *ars1* cluster (*arsR1B1C1H1*) and *ars2* cluster (*arsR2B2C2H2*) scattered on the chromosome (Fig. 1a). The genes in each *ars* cluster were separated by a short sequence of only a few nucleotides, suggesting they were organized in the same operon. To justify this hypothesis, we performed RT-PCR experiments using primers across intergenic regions (Table S3). The results indicate that the genes *arsRBC* within the *arsRBCH* cluster were organized as a co-transcribed operon, whereas *arsH* was in another operon (Fig. 1b).

The degree of DNA sequence identity between homologous genes underscores the appreciable differences between these two *ars* clusters. Specifically, as shown in Fig. S2, *arsR1* and *arsR2* shared 50% sequence identity, *arsB1* and *arsB2* shared 75%, *arsC1* and *arsC2* shared 60%, and *arsH1* and *arsH2* shared 70%. Moreover, two *arsC*-like genes with just 25% homology (*arsC1*-like and *arsC2*-like) were found in the genome. It is an exceptional circumstance that IMH contains two *ars* systems and two As resistance molecular bases, considering that most bacteria have just one such cluster^11^. Therefore, we were motivated to investigate the functional contributions of each *ars* resistance system and molecular base to the As resistance in *Pantoea* sp. IMH.

### Contribution of two *ars* systems and two *arsC*-like genes to As resistance

**Figure 2:**
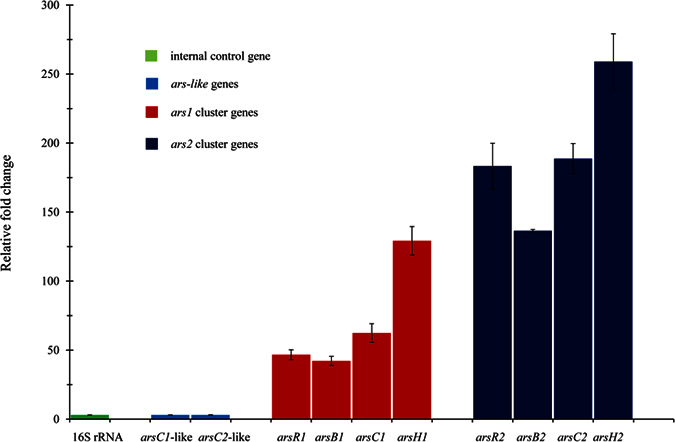
Transcription level of *ars* and *arsC*-like genes of strain IMH in the presence of As(V) with a concentration of 1 mM by RT-Q-PCR. The 16 S rRNA gene was used as an endogenous non-changing control. Data are shown as the means of three replicates, with the error bar illustrating one standard deviation.

We first examined the transcription levels of *ars* genes in each *ars* operon and *arsC*-like gene by performing reverse transcription quantitative PCR (RT-Q-PCR), using 16 S rRNA as an internal control. As shown in Fig. 2, all genes of *ars1* and *ars2* clusters were completely transcribed. Notably, *ars2* genes exhibited about 2–4 fold higher transcription levels than *ars1* genes.

On the other hand, *arsC1*- and *arsC2*-like genes resulted in almost no expression (Fig. 2), indicating that these genes do not contribute to the As resistance. To justify this conclusion, we further analyzed the residues of ArsC in IMH. In contrast to previous observations that four residues of ArsC (Cys-12, Arg-60, Arg-94, and Arg-107) are required for As resistance^12^, Arg-60 and Arg-107 were not conserved in ArsC1-like and ArsC2-like proteins, respectively, in IMH (Fig. S3).

**Figure 3:**
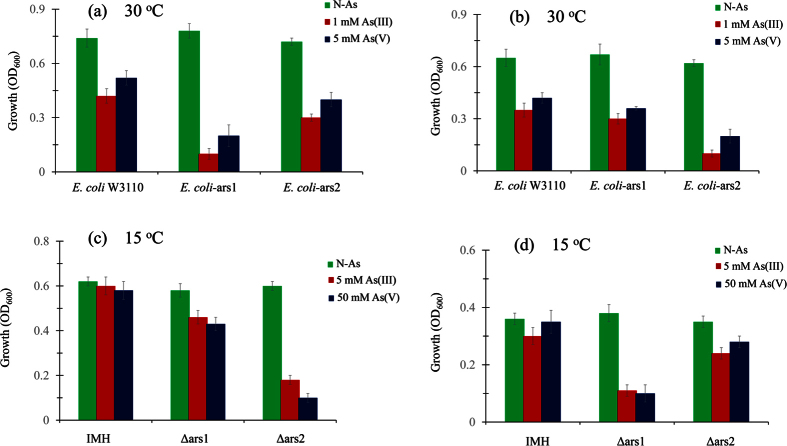
As resistance capability of recombinant bacteria (*E. coli*-ars1 and *E. coli*-ars2) and *ars* cluster deleted strains (Δars1 and Δars2) at 30 °C and 15 °C. Growth of strains for 12 h in stationary phase in LB medium, tested with As(V) and As(III), *E. coli* W3110 and *Pantoea* sp. IMH were used as controls, respectively. IMH: wild type *Pantoea* sp. IMH; Δars1: *ars1* cluster mutant strain; Δars2: *ars2* cluster mutant strain; *E. coli*-ars1: *E. coli* W3110 with recombinant plasmid pUC18-ars1; *E. coli*-ars2: *E. coli* W3110 with recombinant plasmid pUC18-ars2. **(a)** Growth of *E. coli*-ars1 and *E. coli*-ars2 at 30 °C. **(b)** Growth of *E. coli*-ars1 and *E. coli*-ars2 at 15 °C. **(c)** Growth of Δars1 and Δars2 at 30 °C. **(d)** Growth of Δars1 and Δars2 at 15 °C. Data are shown as the means of three replicates, with the error bar illustrating one standard deviation.

To further identify the role of each *ars* cluster, we generated strains Δars1 and Δars2 lacking *ars1* and *ars2* clusters, respectively, based on the genome of IMH as described in the Methods section. The growth of Δars1, Δars2, and the wild type strain IMH was then monitored in the LB medium with 50 mM As(V) and 5 mM As(III). As shown in Fig. 3c, the growth of both Δars1 and Δars2 was substantially suppressed compared to the wild type strain IMH. Specifically, the deletion of *ars1* resulted in more suppression of As resistance than that of *ars2*, implying that *ars2* contributes to a greater extent to the overall As resistance. Moreover, we constructed the functional complementary plasmids pLGM1-ars1 and pLGM1-ars2 (see Methods for details) and then introduced them into the Δars1 and Δars2 strains, respectively. As shown in Fig. S4, the As resistance capabilities of complementary strains Δars1/pLMG1-ars1 and Δars2/pLMG1-ars2 were appreciably improved compared with the deleted strains (Δars1 and Δars2), which confirms the origin of As resistance in the corresponding operons.

Heterologous expression experiments were carried out to study the functional contributions of the two *ars* clusters to the As resistance. Recombinant plasmids for expression of *ars1* and *ars2* clusters were constructed according to the procedure described in the Methods. These plasmids were separately introduced in *E. coli* AW3110 (lacking any As resistance system), yielding the recombinant *E. coli* AW3110-ars1 and *E. coli* AW3110-ars2 strains. The growth of these strains together with the wild type strain *E. coli* W3110 (containing one *ars* operon) was then examined in LB medium containing 5 mM As(V) and 1 mM As(III). The results show that the heterologous host with the *ars2* system acquired a higher resistance to As than that with the *ars1* system (Fig. 3a).

In sum, the two *ars* clusters of strain IMH together contribute to its As resistance, in which the *ars2* cluster is the major contributor. This observation raises a follow-up question: why did *Pantoea* sp. IMH evolve two *ars* systems to resist As? When bacteria species survive under changing environmental circumstances, some proteins may not function under all physicochemical conditions. Bacteria may meet this challenge by having two or more copies of genes to realize the same function under different conditions^13^. An example includes strain *Pseudomonas putida* KT2440, possessing two copies of equivalent *ars* operons to expand its functional scope^14^. Similarly, we propose that possessing two *ars* systems is an evolved strategy for IMH to survive in different ecological niches. To validate our speculation, the function of these two *ars* systems in different ecological conditions was further investigated.

### Functioning of two *ars* systems under different environmental conditions

Major environmental factors, including concentrations of As(V) and As(III), pH, and temperature, may affect As resistance. We therefore tested the performance of each *ars* system in IMH at different pH values (pH 5, 7, and 9), As(V) concentrations (1 and 10 mM), As(III) concentrations (1 and 10 mM), and temperatures (15 and 30 °C). We first examined the transcriptional levels of *arsC1* and *arsC2* using RT-Q-PCR as proxies for the expression of the *ars* systems. Figure S5 shows that the transcription level of *arsC2* was higher than that of *arsC1* for different pH values and concentrations of As(V) and As(III). In contrast, an opposite result (i.e., *arsC1* > *arsC2)* was obtained at a lower temperature (15 °C). This observation suggests that pH, As concentration, and speciation have no influence on the major contribution of *ars2* to As resistance, but a low environmental temperature enables *ars1* to be the predominant contributor.

Furthermore, the growth of *E. coli* AW3110-ars1, *E. coli* AW3110-ars2, Δars1, and Δars2 in LB medium with As at 15 °C was tested, where *E. coli* W3110 and IMH were used as controls. Figure 3b,d shows that *E. coli*-ars1 and Δars2 grew better than *E. coli*-ars2 and Δars1, respectively, at 15 °C, contrary to the results at 30 °C (Fig. 3a,c). In agreement with the RT-Q-PCR results, the above observations confirm that *ars1* contributed more than *ars2* at the lower temperature (15 °C).

**Figure 4:**
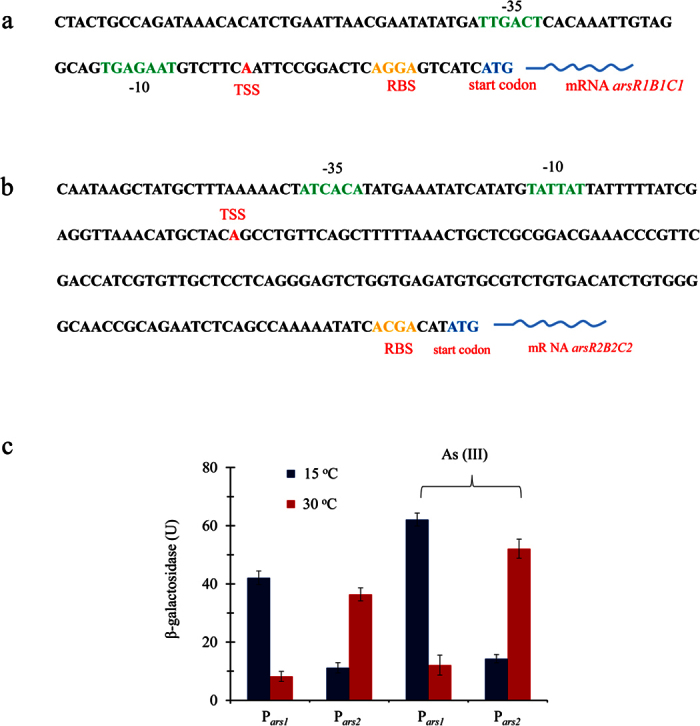
Activity of *P*_*ars1*_and *P*_*ars2*_promoters with transcriptional *lacZ* fusions in *E. coli* AW3110. **(a,b)** The organizations of *P*_*ars1*_ and *P*_*ars2*_. −35 and −10 sequences are marked in green, transcription start sites (TSS) in red, RBSs in yellow, and start codons (ATG) in blue. (**c)** β-galactosidase activity driven by *P*_*ars1*_and *P*_*ars2*_promoters. Cultures were grown in LB medium at either 15 °C or 30 °C in the presence or absence of 1 mM As(III). Data are shown as the means of three replicates, with the error bar illustrating one standard deviation.

Why is this phenotype endowed with two *ars* systems dominant at different temperatures? We hypothesized that the activity of the *arsR* promoter should regulate its expression at different temperatures. To test this hypothesis, we first determined the transcription start site (TSS) using 5′-RACE, and predicted the −35 and −10 regions, as well as ribosomal binding site (RBS) sequence, of each of the promoter regions of *arsR* using SoftBerry software. As shown in Fig. 4a,b, the TSS was located 21 bp upstream of the translational start site of *arsR1* in *ars1*, and 136 bp in *ars2*. The distance between the −10 region and start codon was 28 bp in the *arsR1* promoter and 164 bp in the *arsR2* promoter. The pronounced difference in the organization of these two *arsR* promoters may contribute to their different functions.

To examine the activity of the *arsR* promoter at different temperatures, we assembled equivalent reporter gene fusions between the predicted promoter regions of each *ars* cluster and a *lacZ* reporter gene without its promoter, producing plasmids pPR9TT-P*ars1* and pPR9TT-P*ars2* according to the procedure described in the Methods. The two constructed plasmids were transferred into *E. coli* AW3110, and their β-galactosidase levels were measured at 15 and 30 °C in LB media with and without As(III) (1 mM), which is the effective trigger for the *ars* operon. As shown in Fig. 4c, at the higher temperature (30 °C), the β-galactosidase activity of pPR9TT-P_*ars2*_ was higher than that of pPR9TT-P_*ars1*_ with and without As(III) induction. In contrast, the activity of P_*ars2*_ was noticeably inhibited, while P_*ars1*_ showed comparatively high activity with and without As(III), even higher than that of P_*ars2*_ at the low temperature (15 °C). These results explain the different behaviors of *ars1* and *ars2* expression regarding their temperature dependence. The different performance of the two *ars* promoters was one of the important factors influencing the function of the two *ars* clusters in response to different temperatures. On this basis, we propose that the evolutionary reason for maintaining two *ars* systems in IMH is that their combination facilitates the survival of this strain over an extended range of temperatures in arsenic-polluted niches. This result raises a further question: what is the evolutionary origin of these two *ars* systems and two *arsC*-like genes?

### Evolution of *ars* clusters and *arsC*-like genes in *Pantoea* sp. IMH genome

Substantial differences were observed between the homologous genes, *ars1* and *ars2*, in their sequence identities and As resistance capabilities. We proposed that the differential origins of the two *ars* clusters may have derived from HGT. To validate our speculation, we first compared the genome of IMH to those of other bacterial strains including *P. agglomerans* Tx10, *P. ananatis* LMG20103, *P. dispersa* EGD AAK13, *P. rwandensis* ND04, *P. stewartii* DC283 and *P. vagans* C9-1. A summary of the features for each genome is shown in Table S4. Notably, *ars* genes exist only in *Pantoea* sp. IMH, *P. agglomerans* Tx10, and *P. ananatis* LMG, which belong to a pan-genome (Fig. S6). This result suggests that the *ars* systems in *Pantoea* strains may have been acquired by HGT.

Furthermore, we identified 25 IS elements in the genome of IMH (Table S5) using the IS finder database (http://www-is.biotoul.fr/), and found that some IS elements exist in the flanking region of *ars* clusters (Fig. S7). The IS elements are responsible for transferring genetic information between different cells^15^, and their abundance is positively correlated with HGT^16^.

Moreover, the variation of G + C content between clusters and the genome can be used as an indicator for HGT^17^. Compared to the G + C content of the IMH genome (54.74%), *ars1* (56.28%) and *ars2* (51.86%) clusters exhibited a great difference, supporting the HGT hypothesis.

**Figure 5:**
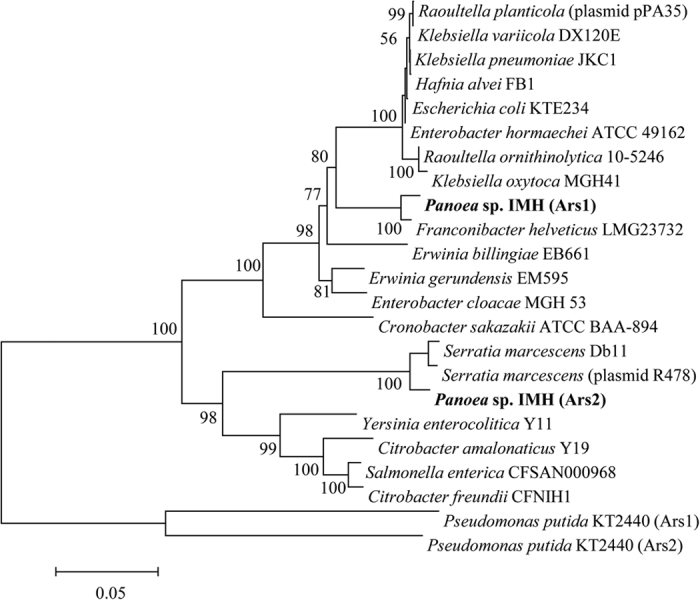
Neighbor-joining tree based on ArsRBCH protein sequences of IMH and representative microorganisms. The numbers at the nodes indicate levels of bootstrap support (%) based on analysis of 100 assembled datasets. Only values at or above 50% are given, bar 0.1 substitutions per amino acid position.

In addition, the phylogenies constructed based on the sequences of ArsRBCH showed that *ars1* and *ars2* clusters of IMH have a sister-group relationship with the *ars* clusters of *Franconibacter helveticus* LMG23732 and *Serratia marcescens* (plasmid R478), respectively (Fig. 5). This result suggests that the *ars1* cluster may originate from a common ancestor with *F. helveticus* LMG23732, and *ars*2 from *S. marcescens* (plasmid R478).

Phylogenies derived from each of the individual ArsR, B, C, and H were congruent with the phylogeny of the concatenated ArsRBCH (Fig. S8–11). Our result is in agreement with previous reports, where microorganisms can obtain the same functional genes from different sources^18^.

To evaluate the evolution of the two *arsC*-like genes, we constructed phylogenetic trees with ArsC and ArsC-like sequences. The result clearly shows that ArsC-like sequences were clustered together themselves and divergent from the sequence of ArsC (Fig. S12). Homology between *arsC* and *arsC*-like genes showed that DNA sequence identity between *arsC1* and *arsC1*-like was 46%, and *arsC2* and *arsC2*-like was 42%, suggesting that the *arsC*-like genes may have resulted from the duplication of *arsC*. Such a phenomenon is not rare in bacterial evolution^19^.

## Discussion

*Pantoea* sp. IMH has been reported to have high resistance to As^10^. However, the molecular mechanism remains unknown. Our study determined that IMH has evolved two different As resistance systems, *ars1* and *ars2* operons, by HGT. At higher temperature (30 °C), the *ars2* operon plays the major role in As resistance, but its function is inhibited at lower temperature (15 °C), where the *ars1* system takes on most of the As resistance function. This kind of genetic constitution for As resistance in IMH is unique compared with other *Pantoea* strains whose genomes have been recently sequenced and annotated. More than one As resistance system in a strain can elevate its As resistance, which explains why IMH can survive in such high As concentrations.

Our genome sequencing showed that there are two *arsC*-like genes in the genome of IMH. Transcription levels of the two *arsC*-like genes were not detected when the strain encountered As (Fig. 2). This result suggests that *arsC*-like genes did not contribute to the As resistance. It is rare for *arsC*-like genes to show no As resistance capability. We further investigated the reasons for this phenomenon. It was reported that Cys-12, Arg-60, Arg-94, and Arg-107 were four conserved residues of the ArsC protein in the process of As resistance^20^. Cys-12 was identified as a catalytic residue and was activated by nearby residues Arg-60, Arg-94, and Arg-107^12^. Alignment analysis showed that Cys-12 and Arg-94 residues were conserved, but residues Arg-107 and Arg-60 in two ArsC-like proteins were not conserved respectively (Fig. S3). These changes in the amino acid sequence further verified that the two ArsC-like proteins did not contribute to As resistance. Interestingly, using phylogenetic analysis, we found that *arsC*-like sequences fell into distinct groups when compared to *arsC* genes. This suggests that multiple *arsC*-like genes may have resulted from *arsC* duplication and had already evolved with deviance. To clarify their relations with As resistance, the structures of ArsC-like proteins are worthy of further study.

It is thought that variants of a core *arsRBC* operon are common in the genomes of various bacteria, and it is rare that more than one *ars* operon appears in the same genome. Bacterial species usually adapt to changing environments by evolving two or more copies of genes, each one performing the same function under differential conditions^13^. Thus, the composition and gene distribution of a genome usually reflect the capacity for adaptation to different ecological niches^14^. In the IMH strain, we indeed found two *ars* systems with different patterns of expression and efficiency at different temperatures. This is a strategy for strain IMH to expand the scope of the encoded function to a wider range of physicochemical settings. Interestingly, our result was consistent with the report on *Pseudomonas putida* KT2440^14^. We speculate that temperature is the most important environmental factor in the evolutionary history of *ars* clusters. Of course, if more strains with two or more copies of *ars* clusters are found, further mechanistic research should be carried out to support this hypothesis.

HGT is an important adaptation strategy to efficiently obtain ‘alien’ DNA^21^. To readily adapt to diverse and stringent growing conditions, IMH obtained two *ars* clusters by HGT. To identify the transfer of genetic information between genomes, we applied three commonly used methods including identification of IS elements and deviant G + C content, and phylogenetic analysis^16^,^19^. The existence of IS elements in the flanking region of *ars* clusters (Fig. S7) together with the greatly different G + C content in the IMH genome (54.74%), *ars1* (56.28%) and *ars2* (51.86%) clusters, suggests that the two *ars* clusters (*arsR1B1C1H1* and *arsR2B2C2H2*) may have been acquired by HGT. Phylogenetic analysis further revealed that the *ars1* cluster may have been acquired via HGT from a source related to *Franconibacter helveticus* LMG23732 in its early evolution, and the *ars2* cluster from *Serratia marcescens* (plasmid R478). Our result is in agreement with previous reports where microorganisms can obtain the same functional genes from different sources^18^.

## Methods

### Genome sequencing, genome annotation and analysis

The genome of strain IMH was sequenced using the IlluminaHiSeq 2000 sequencing platform at the Beijing Genomics Institute (BGI) (Shenzhen, China). Genes were predicted from the assembled result using Glimmer 3.02^22^. The rRNA and tRNA genes were identified with RNAmmer and tRNAscan-SE^23^, respectively. Genome annotation was accomplished by analyzing protein sequences. The resulting translations were aligned with databases, including KEGG 59^24^, GO 1.419^25^ and Swiss-Prot 201206^26^. The draft genome has been deposited in GenBank and the accession number used is JFGT01000000.

### Strains, plasmids and culture conditions

The stains and plasmids used in this work are summarized in Table S2. *E. coli* and *Pantoea* strains were grown in LB medium (per liter contains: 10 g tryptone, 5 g yeast and 10 g NaCl) or LB plates (LB medium with w/v 1.5% agar) at either 15 °C or 30 °C as indicated in each case. When appropriate, antibiotics were added in the following concentrations: 100 μg/mL ampicillin, 100 μg/mL kanamycin, and 100 μg/mL streptomycin. For testing of minimal inhibitory concentrations (MICs), strains were incubated in LB medium with a series of concentrations of As(V) and As(III) as shown in Fig. S1. For monitoring the growth of *E. coli* AW3110-*ars1, E. coli* AW3110-*ars2* and *E. coli* AW3110, strains were cultured in LB medium with 5 mM As(V) and 1 mM As(III) in 96-microwell plates at either 15 °C or 30 °C, and OD_600_ was evaluated at 12 h. For monitoring the growth of Δars1, Δars2 and IMH, strains were cultured in LB medium with 50 mM As(V) and 5 mM As(III) in 96-microwell plates at either 15 °C or 30 °C, and OD_600_ was evaluated at 12 h. When detecting the As resistance under differential pH conditions, the pH of the LB medium was adjusted to pH 5.0, 7.0 and 9.0.

### Construction of recombinant plasmids for expression in *E. coli*

In order to construct the plasmids used in the heterologous expression experiments, genomic DNA of *Pantoea* sp. IMH was used as a template for cloning the two *ars* clusters. A 3.9 kb *Bam*HI-*Xba*I DNA fragment containing the complete *ars1* cluster (promoter region, 360 bp upstream of the start codon ATG of *arsR*, the contiguous four genes *arsR1B1C1H1* and 310 bp upstream of the start codon ATG of *arsH*) was PCR-amplified with primers Ars1-F and Ars1-R (Table S3). A 3.6 kb *Bam*HI-*Xba*I DNA fragment containing the complete *ars2* cluster (a 301 bp region downstream of the stop codon TAA of *arsR2* and the contiguous ten genes *arsR2B2C2H2* and 361 bp downstream of the stop codon TAA of *arsH2*) was PCR-amplified with primers Ars2-F and Ars2-R (Table S3). The PCR products were ligated to the *Bam*HI-*Xba*I site of plasmid pUC18, yielding plasmids pUC18-ars1 and pUC18-ars2. Then the plasmids were transferred to *E. coli* AW3110, yielding the recombinant *E. coli* AW3110-ars1 and *E. coli* AW3110-ars2 strains, respectively.

### Construction of Δars1 and Δars2

To obtain the deleted mutants of *ars1* and *ars2* clusters in *Pantoea sp.* IMH, the suicide vector pARS10 was constructed by inserting the Invitrogen Gateway *attR-Cm*^*R*^ cassette into the backbone of *Sma*I-*Sma*I digested plasmid pKNG101, where *E. coli* DH5α (λpir) was used as the host of pARS10. The Δars1 and Δars2 mutated stains were created using a modified Gateway method described by Choi^27^. To delete the *ars1* cluster, the flanking regions of the *ars1* cluster were amplified by PCR using primers as summarized in Table S3. A kanamycin resistance cassette derived from plasmid pKD4 was inserted between the flanking regions of the *ars1* cluster using a PCR overlap technique with the primers in Table S3. The resulting PCR products containing the Km-resistance cassette flanked by *ars1* cluster were cloned into the Gateway Entry vectors pDNOR221. The construct was transferred into the suicide vector pARS10, obtaining plasmid pARS1-1. The plasmid pARS1-1 was transferred into *E. coli* S17-1 and conjugally introduced into *Pantoea sp.* IMH. An allelic replacement event was selected based on double resistance. PCR with primers listed in Table S3 was used for the verification of the allelic replacement. Generation of the Δars2 strain followed the same method.

### Construction of plasmids for complementation studies

In order to verify the As resistance function of the two *ars* clusters, plasmids pLGM1-ars1 and pLGM1-ars2 were constructed for complementation studies. As described in the previous section titled “Construction of recombinant plasmids for expression in *E. coli*”, *ars1* and *ars2* were amplified and then ligated to the *Bam*HI- *Eco*RI site of plasmid pLGM1, yielding pLGM1-ars1 and pLGM1-ars2.

### RT-PCR analysis

In order to determine the operons in *ars* clusters, an RT-PCR experiment with primers designed to span across intergenic regions (Table S3) was carried out. A culture of *Pantoea sp.* IMH was grown in LB medium with 1 mM As(V). After 8 h, the IMH strains were harvested by centrifugation at 4 °C, and the total RNA was isolated using the PrimeScript^®^ RT reagent Kit with gDNA Eraser (Takara Bio) according to the manufacturer’s instructions. The possibility of contamination of genomic DNA was eliminated by digestion with RNase-free DNase I (Takara Bio). The integrity and size distribution of the RNA were verified by agarose gel electrophoresis, and the concentration was determined spectrophotometrically. Synthesis of cDNA was carried out using RT Prime Mix according to the manufacturer’s specification (Takara Bio). 1.0 μg of cDNA was used for the template of RT-PCR.

### RT-Q-PCR analysis

In order to understand the differences in each gene’s transcription level in *ars1* and *ars2* clusters under differential environmental factors, RT-Q-PCR analysis was used. *Pantoea sp.* IMH was grown in LB medium with different As(V) or As(III) concentrations (1 mM and 10 mM), in LB medium with 1 mM As(V) at different temperatures (15 °C and 30 °C), and in LB medium with different pH (5, 7 and 9). Then the cDNA was obtained as described in the RT-PCR analysis. Specific cDNA was employed to quantify the transcriptional signals of the *ars* genes and *arsC-*like genes, where 16 S rRNA gene was used as an internal reference. Primers used are listed in Table S3. RT-Q-PCR reactions were performed with three replicates using the ABI applied Biosystems vii A7.

### Transcription start site identification

To determine the transcription start site (TSS) of the two *ars* operons, the 5′-RACE method was employed using the SMARTer™ RACE cDNA Amplification Kit (Clontech). Gene-specific primers are listed in Table S3. The PCR product was cloned into the pMD18-T Vector and then sequenced.

### P_*ars*_-*lacZ* transcriptional fusions and β-galactosidase assays

To explain the reason for the distinctly different performance of *ars1* and *ars2* clusters at 15 °C and 30 °C, the promoter activities of the two *ars* clusters were determined. The promoter of the *ars1* cluster - a 110 bp DNA fragment (P_*ars1*_) (from −107 to +3 relative to the *arsR1* transcription start codon) - and the promoter of the *ars2* cluster - a 241 bp DNA fragment (P_*ars2*_) (from −238 to +3 relative to the *arsR2* transcription start codon) - were amplified from the total DNA of *Pantoea sp.* IMH using primers listed in Table S3. The amplified fragments were then cloned into the promoter vector pPR9TT, generating transcriptional fusions between the inserted promoter regions and a promoterless, complete *lacZ* gene, pPR9TT-P_*ars1*_and pPR9TT-P_*ars2*_. The plasmids were then transformed into *E. coli* DH5α, yielding *E. coli* DH5α/P_*ars1*_::*lacZ* and *E. coli* DH5α/P_*ars2*_::*lacZ*, respectively. For β-galactosidase activity assays, strains *E. coli* DH5α/P_*ars1*_::*lacZ* and *E. coli* DH5α/P_*ars2*_::*lacZ* were grown in LB medium with 1 mM As(III) for 12 h at 15 °C and 30 °C with shaking. β-galactosidase activity was measured according to the method described by Miller^28^. Briefly, a 100 μL sample was mixed with 900 μL Z buffer and shaken for 20 sec. Then, 200 μL o-nitrophenyl-β-D-galactopyranoside (ONPG) (4 mg/mL) was added and incubated for 20 min at 30 °C. To stop the above reaction, 500 μL of 1 M Na_2_CO_3_ solution was used. Finally, the OD_420_ and OD_550_ values were measured after the mixture was centrifuged. β-galactosidase (1 unit) = [1000 × (OD_420_ − 1.7 × OD_550_)]/[Time (min) × Vol (mL) × OD_600_].

### Comparative genomics

All orthologous pairs between tests of *Pantoea* genomes were identified by Pan Genome Analysis Pipeline^29^. The common dataset of shared genes among test strains was defined as their core genome. The total set of genes with test genomes was defined as the pan-genome. The unshared genes in each strain were defined as unique genes. The genomes used in this study are listed in Table S4.

### Phylogenetic analysis

In order to analyze the evolution of the two *ars* clusters of strain IMH, phylogenetic trees were constructed using the neighbor-joining method. Evolutionary distances were calculated according to Kimura’s two-parameter model. Bootstrap analysis was performed on the basis of 1000 replications. The software package MEGA version 5.0 was used.

## Additional Information

**How to cite this article**: Wang, L. *et al*. Arsenic resistance strategy in *Pantoea* sp. IMH: Organization, function and evolution of *ars* genes. *Sci. Rep.* **6**, 39195; doi: 10.1038/srep39195 (2016).

**Publisher’s note:** Springer Nature remains neutral with regard to jurisdictional claims in published maps and institutional affiliations.

## Supplementary Material

Suport Information
