# Supplementary material for: Arsenic resistance strategy in Pantoea sp. IMH: Organization, function and evolution of ars genes
Source: Sci Rep. 2016 Dec 14;6:39195. doi: 10.1038/srep39195 (PMC5155266; doi:10.1038/srep39195)
Supplement: Suport Information [file srep39195-s1.pdf]

Supporting Information

Arsenic resistance strategy in *Pantoea* sp. IMH: Organization, function and evolution of *ars* genes

Liying Wang[a,b], Xuliang Zhuang[a,b], Guoqiang Zhuang[a,b], Chuanyong Jing[a,b] *

[a]State Key Laboratory of Environmental Chemistry and Ecotoxicology, Research Center for Eco−Environmental Sciences, Chinese Academy of Sciences, P.O. Box 2871, Beijing 100085, China.

[b]University of Chinese Academy of Sciences, Beijing 100049, China

***Corresponding author:** Dr. Chuanyong Jing; Tel: +86 10 6284 9523;

E-mail: cyjing@rcees.ac.cn

Number of pages: 19

Number of figures: 12

Number of tables: 5

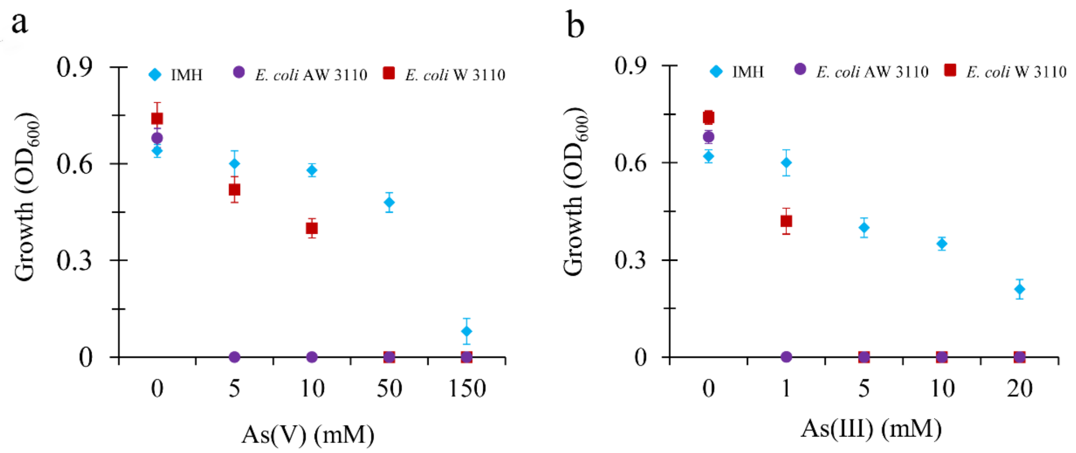

Supplementary Figure S1. Growth of strains *E. coli* AW3110, *E. coli* W3110, and IMH in liquid LB medium with increasing As concentrations.

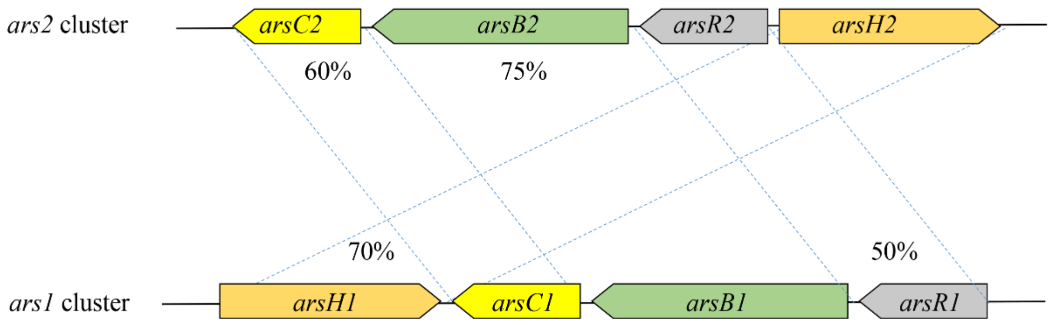

Supplementary Figure S2. Homology analysis of two *ars* clusters based on gene sequences. DNA sequence identity through the homologous regions is 50% (*arsR*), 75% (*arsB*), 60% (*arsC*) and 70% (*arsH*).

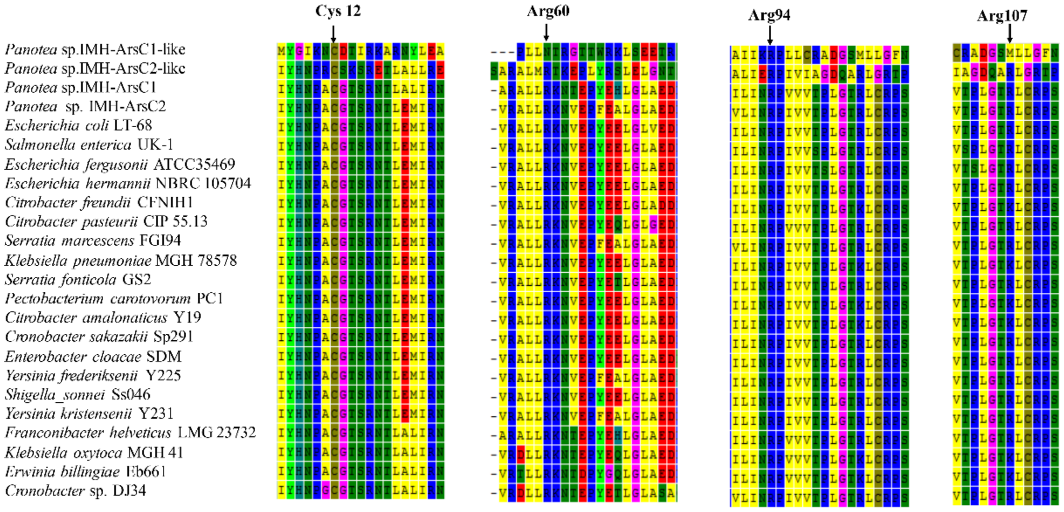

Supplementary Figure S3. **Alignment surrounding crucial residues Cys-12, Arg-60, Arg-94, and Arg-107 in ArsC and ArsC-like protein sequences from *Pantoea* sp. IMH and other As-resistant organisms.** Crucial residues Cys-12, Arg-60, Arg-94, and Arg-107 are indicated with vertical arrows.

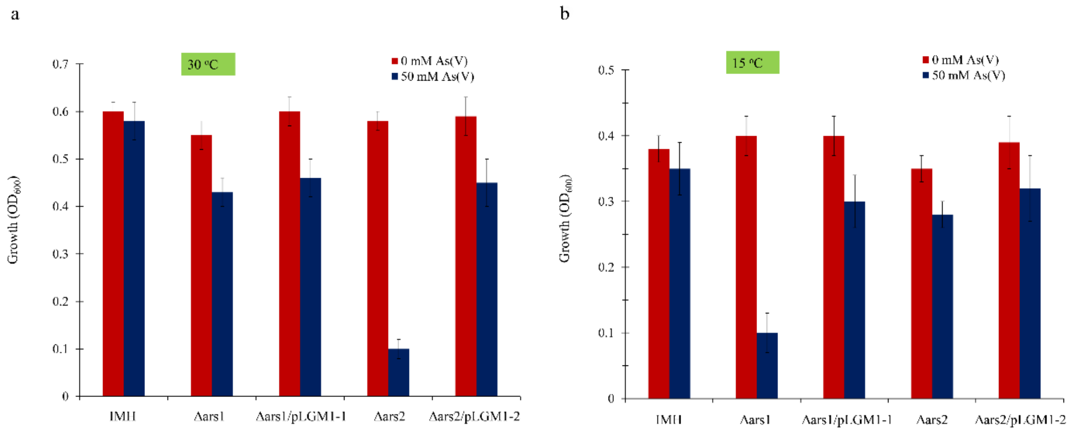

Supplementary Figure S4. **As resistance capability of complementary strains and control strains at 30 °C and 15 °C.** IMH: wild type *Pantoea* sp. IMH; Δars1: *ars1* cluster mutant strain; Δars2: *ars2* cluster mutant strain; Δars1/pLMG1-ars1: Δars1 with plasmid pLMG1-ars1; Δars2/pLMG1-ars2: Δars2 with plasmid pLMG1-ars2.

(a) Growth of strains for 12 h in stationary phase in LB medium with a concentration of 50 mM As(V) at 30 °C.

(b) Growth of strains for 12 h in stationary phase in LB medium with a concentration of 50 mM As(V) at 15 °C.

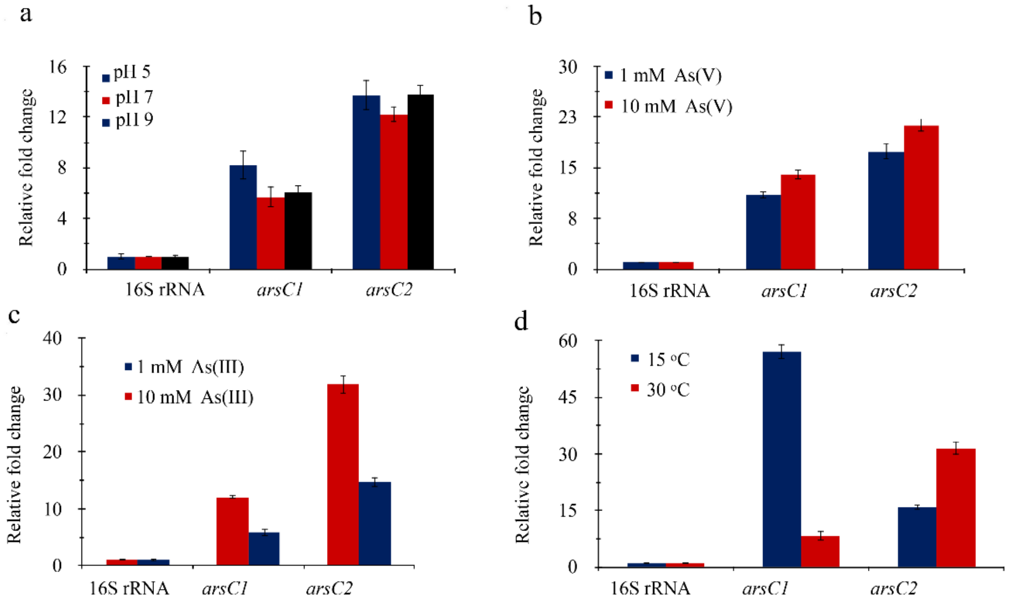

Supplementary Figure S5. Relative expression of *Pantoea* sp. IMH *arsC* genes under different environmental conditions. The 16S rRNA gene was used as an endogenous non-changing control. Data are shown as the mean of three replicates, with the error bars illustrating one standard deviation. (**a)** shows the results at different pH values; (**b)** shows the results in different As(V) concentrations; (**c)** shows the results in different As(III) concentrations and (**d)** shows the results at different temperatures.

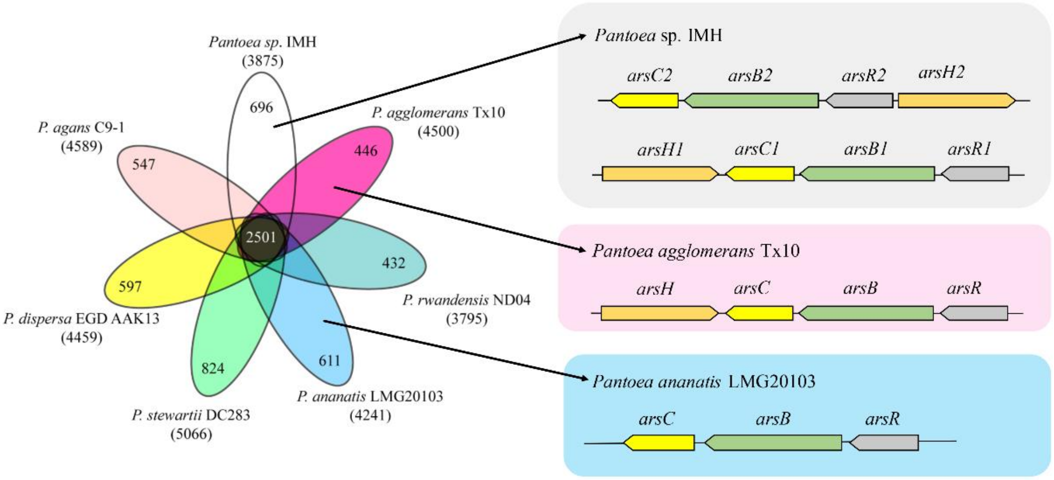

Supplementary Figure S6. *ars* systems in *Pantoea* spp. strains. Comparative genomics analysis of 7 species in genus *Pantoea* showed that *ars* systems were distributed in the pan-genome, and were found only in *Pantoea* sp. IMH, *Pantoea agglomerans* Tx10 and *Pantoea ananatis* LMG20103.

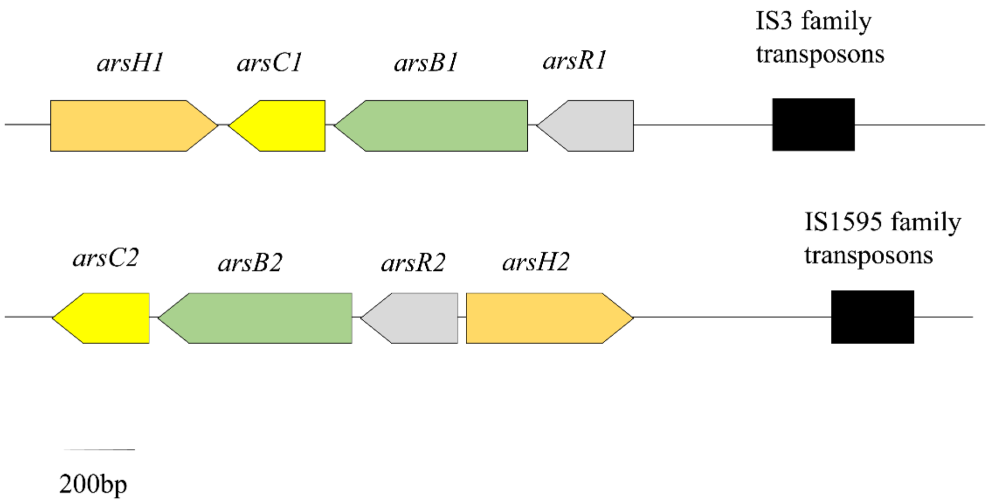

Supplementary Figure S7. ***ars*** **clusters linked to transposon elements.** IS3 family transposon was found to be located on the flanking region of the *ars1* cluster, IS1595 family transposon was found to be located on the flanking region of the *ars2* cluster.

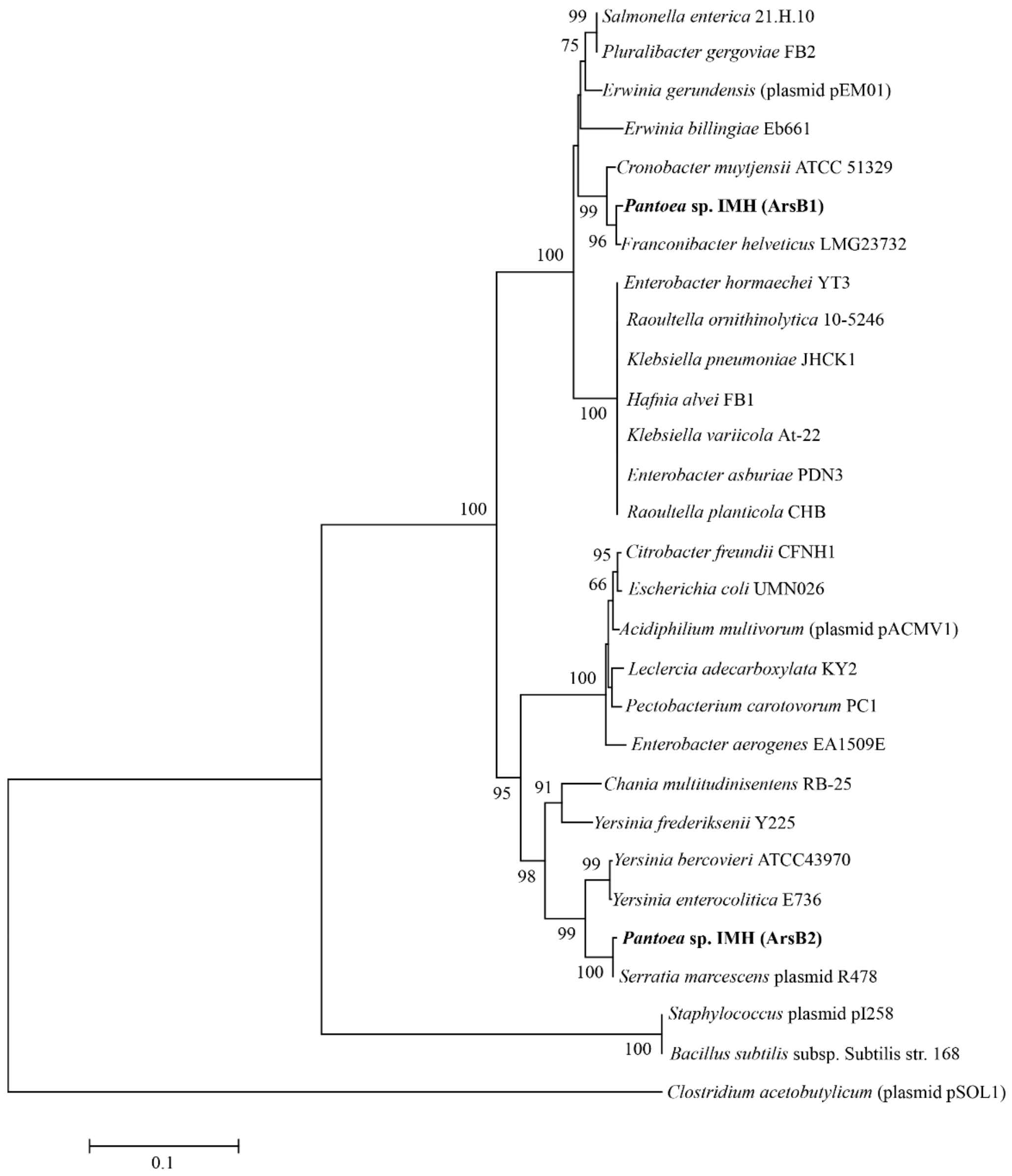

Supplementary Figure S8. Neighbor joining phylogenetic tree of the ArsB protein sequences derived from *Pantoea* sp. IMH and other representative species. A total of 1,000 bootstrap replicates were made, and bootstrap values are indicated at each node.

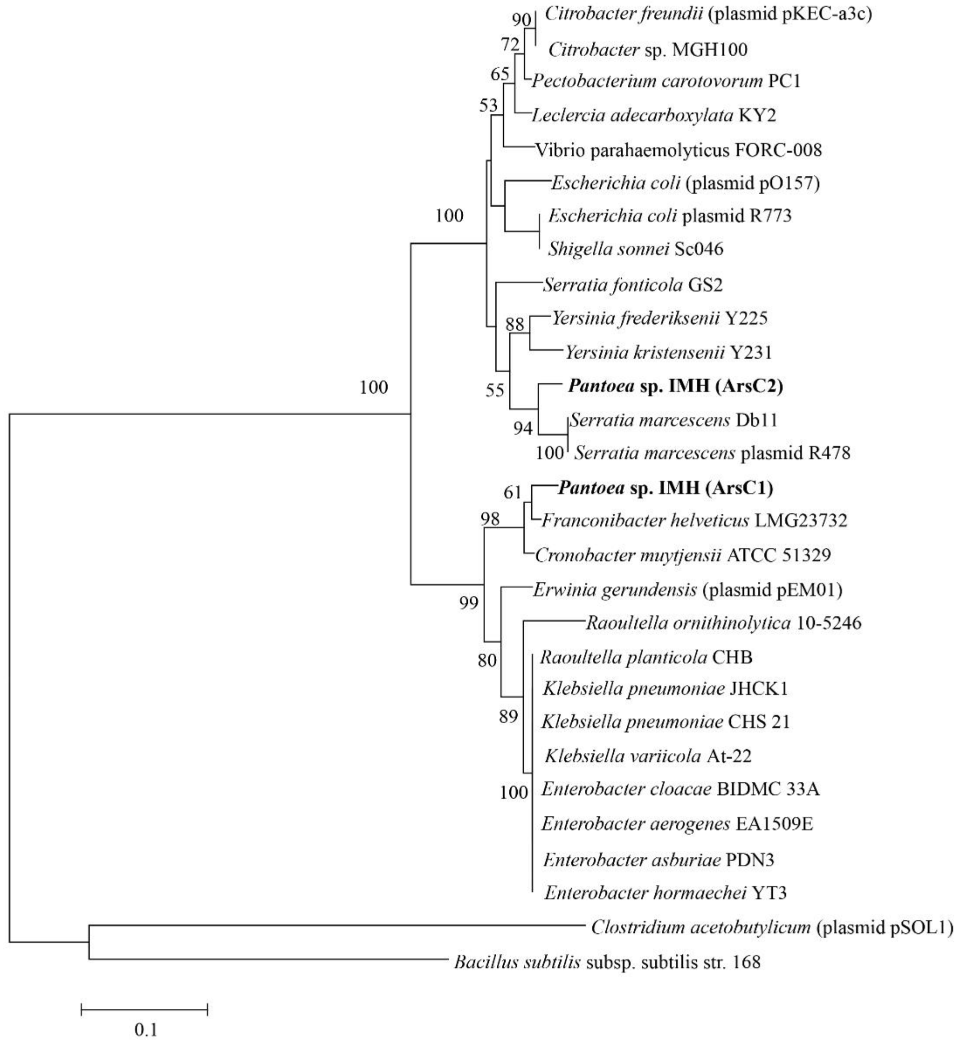

Supplementary Figure S9. Neighbor joining phylogenetic tree of the ArsC protein sequences derived from *Pantoea* sp. IMH and other representative species. A total of 1,000 bootstrap replicates were made, and bootstrap values are indicated at each node.

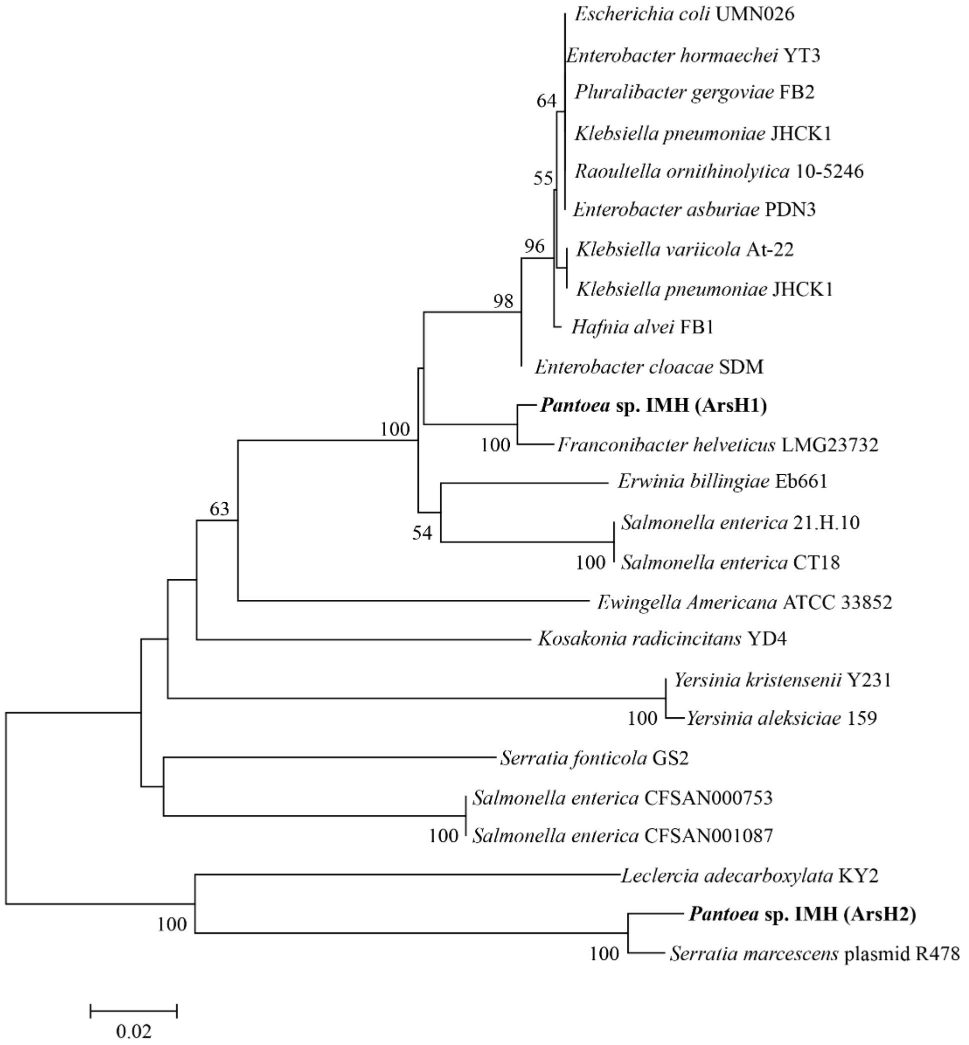

Supplementary Figure S10. Neighbor joining phylogenetic tree of the ArsH protein sequences derived from *Pantoea* sp. IMH and other representative species. A total of 1,000 bootstrap replicates were made, and bootstrap values are indicated at each node.

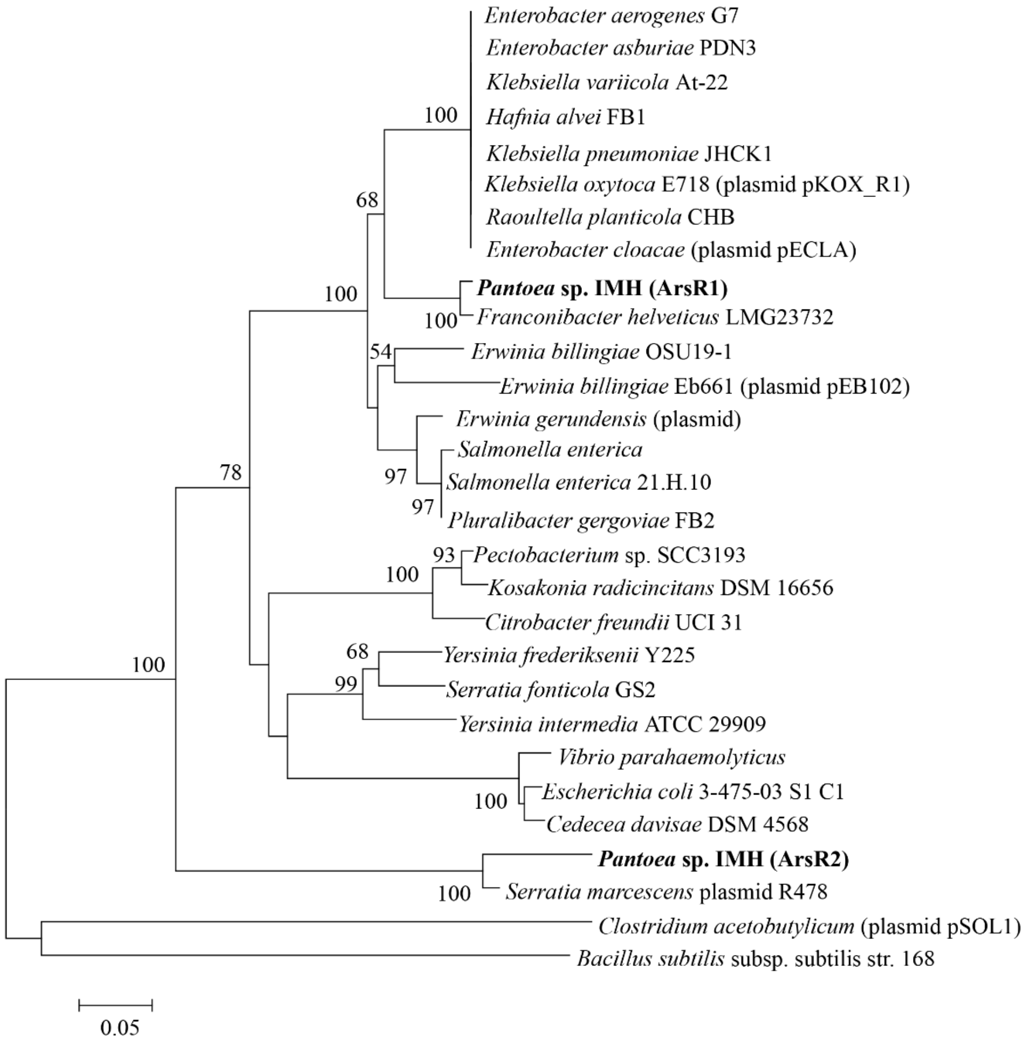

Supplementary Figure S11. Neighbor joining phylogenetic tree of the ArsR protein sequences derived from *Pantoea* sp. IMH and other representative species. A total of 1,000 bootstrap replicates were made, and bootstrap values are indicated at each node.

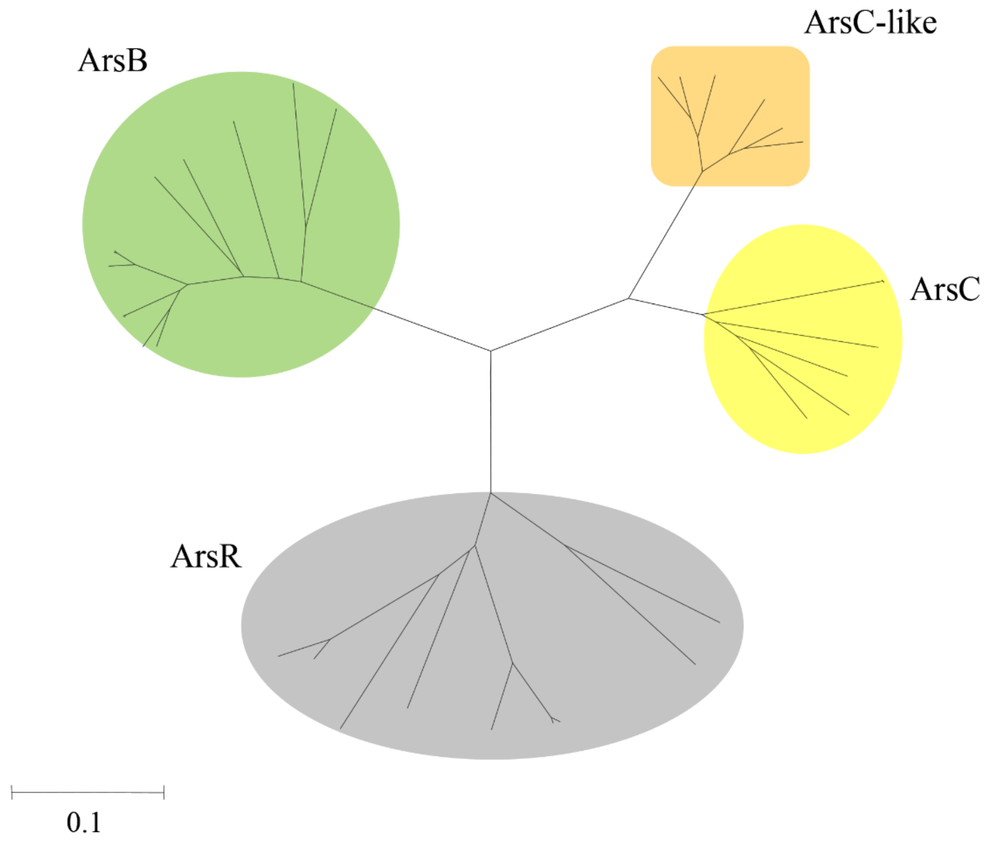

Supplementary Figure S12. Neighbor-joining tree based on ArsRBC/ArsC-like proteins. ArsRBC/ArsC-like sequences were derived from *Pantoea* sp. IMH and the representative microorganisms.

Supplementary Table S1. Overview on the arsenic resistance levels of some strains.

Name	As resistance MIC (mmol l^{-1})		*ars* genes and clusters	Sources
	As(III)	As(V)		
E. coli W3110	1	2-3	*arsCBR*	[1]
Bacillus subtilis	1	4	*arsRBC*	[2]
Staphylococcus aureus	2	50	*arsCBR*	[3]
Thiomonas sp. 3As	6	50	*arsRDABC*, *arsRBC* and *aioAB*	[4]
Geobacillus kaustophilus A1	5	80	*arsCBR* and *arsCBR*	[5]
Herminiimonas arsenicoxydans	5	100	*arsRCBH*, *arsRCBH*, *arsRC-acr3-arsCH and arsRCH*	[6]
Ferroplasma acidarmanus Fer1	67-133	133	*arsRB*	[7]
Acidovorax sp. NO1	20	200	*aioRSXABDC*, *arsHC-acr3-arsR* and *arsRCDACBSB*	[8]
Ochrobactrum tritici SCII24T	50	200	*arsRDAB*, *arsRC-acr3-arsCHR*	[9]
P. putida KT2440	10	300	*arsCBRH* and *arsCBRH*	[10]
Microbacterium sp. A33	28	400	*arsCTX-acr3-arsRC2C1*	[11]
C. glutamicum ATCC 13032	12	500	*arsRBC*, *arsRBCC*, *arsB* and *arsC*	[12]
Pantoea sp. IMH	20	150	*arsRBCH*, and *arsRBCH*	This study

Supplementary Table S2. Strains and plasmids used in this work.

strains	characteristics	sources
IMH	Arsenate reducing bacteria IMH	Our lab
Δars1	Arsenate reducing bacteria IMH with *ars1* deleted	This study
Δars2	Arsenate reducing bacteria IMH with *ars2* deleted	This study
E. coli Top10	F- *mcr*A Δ(*mrr-hsd* RMS-*mcr* BC) Ø 80 *lac*ZΔM15 Δ*lac* X74 *rec*A1 *ara*Δ139 Δ(*ara-leu*) 7697 *gal*U *gal*K *rps*L (Str^R) *end*A1 *nup*G	TaKaRa
E. coli DH5α	F- Φ80 Δ*lacZ* ΔM15 Δ(*lacZYA-argF*) U169 *recA1 endA1 hsdR17* R^-M^+ *supE44 thi gyrA relA*	TaKaRa
E. coli JM109	*recA1*, *endA1*, *gyrA96thi-1*, *hsdR17*, *supE44*, *relA1*, Δ(*lac-proAB*)/F'[*traD36*, *proAB+*, l*acIq*, *lacZΔM15*]	TaKaRa
JM109/pUC18	Derivative of JM109, carrying empty vector pUC18, Amp^R	TaKaRa
E. coli S17-1	RP4 Mob$^+$, Sm^R	[13]
E. coli W3110	K12 F^- IN (*rrnD-rrnE*)	[1]
E. coli C3110	K12 F^- Δ*arsC*::*cam* F^- IN (*rrnD-rrnE*)	[1]
E. coli AW3110	K12 F^- Δ*ars*::*cam* F^- IN (*rrnD-rrnE*)	[1]
**Plasmids**		
pUC18	cloning vector, Amp^R	TaKaRa
pUC18-ars1	Amp^R, pUC18 inserted with PCR fragment spanning complete *ars1* cluster with its native promoter	This study
pUC18-ars2	Amp^R, pUC18 inserted with PCR fragment spanning complete *ars2* cluster with its native promoter	This study
pUC18-arsC1-like	Amp^R, pUC18 inserted with PCR fragment spanning complete *arsC1*-like with its native promoter	This study
pUC18-arsC2-like	Amp^R, pUC18 inserted with PCR fragment spanning complete *arsC2*-like with its native promoter	This study
pPR9TT	9388 bp broad-host-range plasmid carrying promoterless *lacZ* without ATG, Amp^R Cm^R	Sangon Biotech Co
pPR9TT-P*ars1*	pPR9TT inserted with P_{ars1} promoter, Amp^R Cm^R	This study
pPR9TT-P*ars2*	pPR9TT inserted with P_{ars2} promoter, Amp^R Cm^R	This study
pKNG101	Suicide vector, Str^R	[14]
pARS10	Suicide vector, Gateway *attR-Cm^R* cassette clone in pKNG101, Sm^R, Cm^R	This study
pDONR221	Cloning vector, Km^R	
pARS10-1	*ars1*::*Km^R* cloned in pARS10	This study
pARS10-2	*ars2*::*Km^R* cloned in pARS10	This study
pKD4	Km^R	Sangon Biotech Co

pLGM1	A 6.37 kb shuttle vector between *E. coli* and *P. agglomerans*, Amp^R	[15]
pLGM1-ars1	Plasmid pLGM1 inserted with complete *ars1* cluster, Amp^R	This study
pLGM1-ars2	Plasmid pLGM1 inserted with complete *ars2* cluster, Amp^R	This study

Supplementary Table S3. Primers used in this work.

Name	Sequence (5'-3')	Location/Target
Ars1-F	CGGTACCCGGGGATCCTCAATTGTCATAACCCACTCAC	For cloning *ars1* cluster to pUC18
Ars1-R	GCAGGTCGACTCTAGATGATAAGCTTTGTTTCCACC	For cloning *ars1* cluster to pUC18
Ars2-F	CCATGATTACGAATTCCTGGGGCTGCGCGAGAAGTA	For cloning *ars2* cluster to pUC18
Ars2-R	CGACTCTAGAGGATCCGAGAAACCTGGAACGTCTGA	For cloning *ars2* cluster to pUC18
ArsC1-like-F	CGGTACCCGGGGATCCCGGGATGGTCTCTCAGTACC	For cloning *arsC1*-like cluster to pUC18
ArsC1-like-R	GCAGGTCGACTCTAGACTGGCCGCGCCAGGCCCAGAA	For cloning *arsC1*-like cluster to pUC18
ArsC2-like-F	CGGTACCCGGGGATCCATCATGACCGATATTGAGA	For cloning *arsC2*-like cluster to pUC18
ArsC2-like-R	GCAGGTCGACTCTAGAAAGATGTGGGCCGCTTCCT	For cloning *arsC2*-like cluster to pUC18
Pars1-F	ATTCGATATCAAGCTTGAAGTATGATGCAGAACTCCG	For cloning promoter of *ars1* cluster to pPR9TT
Pars1-R	GGGAACAAAAGCTGGGTACCCCTGAGTCCGGAATTGAAGA	For cloning promoter of *ars1* cluster to pPR9TT
Pars2-F	ATTCGATATCAAGCTTATCTTTGCCAGCAACCGTC	For cloning promoter of *ars2* cluster to pPR9TT
Pars2-R	GGGAACAAAAGCTGGGTACCGTCGTGATATTTTTGGCTGAG	For cloning promoter of *ars2* cluster to pPR9TT
ars1-up-F	GTCGACGGATCCCCGGGAGGGGTTCAGAAAGGTCGGTTA	For construction of Δars1
ars1-up-R	CCACTTCTAGGAAAAACTACTTGCCGTCTCGCCTGTCG	For construction of Δars1
kan1-F	AGGGGTTCAGAAAGGTCGGTAGTTTTTCCTAGAAGTGG	For construction of Δars1
kan1-R	AAGCTGAGGGCTGCAGAAGTACGTCGAGACCGGGCACAGAG	For construction of Δars1
ars1-down-F	CTCTGTGCCCGGTCTCGACGTAAGTATCAATATTTTTCAGG	For construction of Δars1

ars1-down-R	ATATGCATCCGCGGGCCCGGGTAGCGCTG ATAAGCTTTGTT	For construction of Δars1
ars2-up-F	GTCGACGGATCCCCGGGTGAACCAGCAC GGCGATATC	For construction of Δars2
ars2-up-R	CCACTTCTAGGAAAAACTAAGGCCCAGAT TGACTGGATA	For construction of Δars2
kan2-F	TATCCAGTCAATCTGGGCCTTAGTTTTTCC TAGAAGTGG	For construction of Δars2
kan2-R	CTCGTGATGGTGGTGTCTCAACGTCGAGA CCGGGCACAGAG	For construction of Δars2
F-ars2-down	CTCTGTGCCCGGTCTCGACGTTGAGACAC CACCATCACGAG	For construction of Δars2
F-ars2-down	ATATGCATCCGCGGGCCCGGGATACTGGA AAGTTGTGCTGA	For construction of Δars2
Ars1-co-F	GACGGAGCTCGAATTCATGCCTCAATTTC AGCCTCTTC	For construction of pLGM1-ars1
Ars1-co-R	AAATGGGTCGGGATCCCTTGAACAGCTG ATGGCCTTTC	For construction of pLGM1-ars1
Ars2-co-F	GACGGAGCTCGAATTCCTGGGGCTGCGC GAGAAGTA	For construction of pLGM1-ars2
Ars2-co-R	AAATGGGTCGGGATCCGAGAAACCTGGA ACGTCTGA	For construction of pLGM1-ars2
arsR1-F	GGAGAGCTTTGCGTCTGTG	RT-PCR and qRT-PCR for *arsR1*
arsR1-R	GACCGGCGTCTCTGAGTAAC	qRT-PCR for *arsR1*
arsB1-F	AGGCTTTATCGCGGACAC	RT-PCR and qRT-PCR for *arsB1*
arsB1-R	TTACCGGCACCATGACAGAC	RT-PCR and qRT-PCR for *arsB1*
arsC1-F	CACCCCATCCTCATCAACCG	RT-PCR and qRT-PCR for *arsC1*
arsC1-R	GCAGAATGTCCAGAACCGC	RT-PCR and qRT-PCR for *arsC1*
arsH1-F	CGTGCCGCTGACTGAAGATAAG	RT-PCR and qRT-PCR for *arsH1*
arsH1-R	TGTGAGACGGCTGTATGAGCG	qRT-PCR for *arsH1*

arsR2-F	GGAAAATGGGTGCACTACCG	RT-PCR and qRT-PCR for *arsR2*
arsR2-R	TCACAGTTCCAGGCCGTATC	RT-PCR and qRT-PCR for *arsR2*
arsB2-F	TAGCGTTTATCATGGCCGC	RT-PCR and qRT-PCR for *arsB2*
arsB2-R	TCCGCCGAGACGATATTCAC	RT-PCR and qRT-PCR for *arsB2*
arsC2-F	ACAGAGCCGACCGTTATTC	RT-PCR and qRT-PCR for *arsC2*
arsC2-R	CGAGATCCCCATATCCGC	RT-PCR and qRT-PCR for *arsC2*
arsH2-F	TGCCGTTACCCGATGATG	qRT-PCR for *arsH2*
arsH2-R	CAGACCATTCCGTCACACC	RT-PCR and qRT-PCR for *arsH2*
arsC1-F	GCAGAAATTTATCGACAAC	qRT-PCR for *arsC1-like*
arsC1-R	TCAGGACTTCTCCATAATAA	qRT-PCR for *arsC1-like*
arsC2-F	AGAACGGTGTGGAGCCGG	qRT-PCR for *arsC2-like*
arsC2-R	TCGCTGATGCTGGTGTTG	qRT-PCR for *arsC2-like*
16S1	TTCGGTCGGGAACTCAAAG	qRT-PCR for control
16S2	GTATGCGCCATTGTAGCACG	qRT-PCR for control
IMH-GSP2	ATCTCCAGATCGCGCAATGCTG	5' RACE
IMH-GSP3	TCATTGATGCACTCGAATATG	5' RACE

Supplementary Table S4. Genomic features of *Pantoea* strains.

Species	INSDC	Size (Mb)	Protein	G+C Content	tRNA genes	rRNA genes	Gene
Pantoea sp. IMH	JFGT00000000	4.09	3875	54.7	81	23	3875
P. agglomerans Tx10	ASJI00000000.1	4.86	4500	55.1	71	24	4627
P. ananatis LMG20103	CP001875.2	4.70	4241	53.7	68	22	4349
P. dispersa EGD AAK13	AVSS00000000.1	4.77	4420	57.8	66	5	4494
P. rwandensis ND04	CP009454.1	4.33	3795	53.9	79	22	3932
P. stewartii DC283	AHIE00000000.1	5.23	4903	53.8	70	20	5182
P. vagans C9-1	CP002206.1	4.02	3664	55.5	77	22	3764

Supplementary Table S5. IS elements in strain *Pantoea* sp. IMH.

Sequences producing significant alignments	Family	Origin
ISBth13	IS110	*Bacillus thuringiensis*
ISNpe19	IS5	*Natrinema pellirubrum*
ISSen3	IS21	*Salmonella enterica*
ISSen4	IS3	*Salmonella enterica*
ISKol9	IS3	*Kosmotoga olearia*
ISEc25	IS3	*Escherichia coli*
ISYen3	IS3	*Yersinia enterocolitica*
ISBce4	IS4	*Bacillus cereus*
ISBth5	IS4	*Bacillus thuringiensis*
ISLpn3	IS4	*Legionella pneumophila*
ISPpu1	IS630	*Pseudomonas putida*
IS1222	IS3	*Enterobacter agglomerans*
ISPath1	IS110	*Pandoraea thiooxydans*
ISApr13	IS5	*Alpha proteobacterium*
ISMaq6	IS5	*Marinobacter aquaeolei*
ISAde5	IS256	*Anaeromyxobacter dehalogenans*
ISAli10	IS66	*Azospirillum lipoferum*
ISBvu2	IS1595	*Bacteroides vulgatus*
ISLpn2	IS4	*Legionella pneumophila*
ISMhu6	IS4	*Methanospirillum hungatei*
ISH16	IS630	*Haloarcula marismortui*
ISAzvi3	IS110	*Azotobacter vinelandii*
ISPsy16	IS110	*Pseudomonas syringae*
ISSc1	IS30	*Spiroplasma citri*
IS1489v1	ISL3	*Pseudomonas putida*

References

1. Carlin, A., Shi, W., Dey, S. & Rosen, B. P. The *ars* operon of Escherichia coli confers arsenical and antimonial resistance. *J. Bacteriol.* **177**, 981-986 (1995).
2. Sato, T. & Kobayashi, Y. The *ars* operon in the *skin*Element of *Bacillus subtilis* confers resistance to arsenate and arsenite. *J. Bacteriol.* **180**, 1655-1661 (1998).
3. Ji, G. & Silver, S. Reduction of arsenate to arsenite by the ArsC protein of the arsenic resistance operon of *Staphylococcus aureus* plasmid pI258. *Proc. Natl. Acad. Sci. USA* **89**, 9474-9478 (1992).
4. Arsène-Ploetze, F. *et al.* Structure, function, and evolution of the *Thiomonas* spp. genome. *PLoS Genet.* **6**, e1000859 (2010).
5. Cuebas, M., Villafane, A., McBride, M., Yee, N. & Bini, E. Arsenate reduction and expression of multiple chromosomal *ars* operons in *Geobacillus kaustophilus* A1. *Microbiology* **157**, 2004-2011 (2011).
6. Muller, D. *et al.* A tale of two oxidation states: bacterial colonization of arsenic-rich environments. *PLoS Genet.* **3**, e53 (2007).
7. Baker-Austin, C. *et al.* Extreme arsenic resistance by the acidophilic archaeon '*Ferroplasma acidarmanus*' Fer1. *Extremophiles* **11**, 425-434 (2007).
8. Huang, Y. *et al.* Genome sequence of the facultative anaerobic arsenite-oxidizing and nitrate-reducing bacterium *Acidovorax* sp. strain NO1. *J. Bacteriol.* **194**, 1635-1636 (2012).
9. Branco, R., Chung, A.-P. & Morais, P. V. Sequencing and expression of two arsenic resistance operons with different functions in the highly arsenic-resistant strain *Ochrobactrum tritici* SCII24T. *BMC Microbiol.* **8**, 95 (2008).
10. Páez-Espino, A. D., Durante-Rodríguez, G. & Lorenzo, V. Functional coexistence of twin arsenic resistance systems in *Pseudomonas putida* KT2440. *Environ. Microbiol.* **17**, 229-238 (2015).
11. Achour-Rokbani, A., Cordi, A., Poupin, P., Bauda, P. & Billard, P. Characterization of the *ars* gene cluster from extremely arsenic-resistant *Microbacterium* sp. strain A33. *Appl. Environ. Microbiol.* **76**, 948-955 (2010).
12. Ordóñez, E., Letek, M., Valbuena, N., Gil, J. A. & Mateos, L. M. Analysis of genes involved in arsenic resistance in *Corynebacterium glutamicum* ATCC 13032. *Appl. Environ. Microbiol.* **71**, 6206-6215 (2005).
13. Simon, R., Priefer, U. & Pühler, A. A broad host range mobilization system for in vivo genetic engineering: transposon mutagenesis in gram negative bacteria. *Nat. Biotechnol.* **1**, 784-791 (1983).
14. Kaniga, K., Delor, I. & Cornelis, G. R. A wide-host-range suicide vector for improving reverse genetics in gram-negative bacteria: inactivation of the *blaA* gene of *Yersinia enterocolitica*. *Gene* **109**, 137-141 (1991).
15. de Lima Procopio, R. E., Araujo, W. L., Andreote, F. D. & Azevedo, J. L. Characterization of a small cryptic plasmid from endophytic *Pantoea agglomerans* and its use in the construction of an expression vector. *Genet. Mol. Biol.* **34**, 103-109 (2011).
